# FGF21 negatively affects long-term female fertility in mice

**DOI:** 10.1016/j.heliyon.2022.e11490

**Published:** 2022-11-11

**Authors:** Beat Moeckli, Thuy-Vy Pham, Florence Slits, Samuel Latrille, Andrea Peloso, Vaihere Delaune, Graziano Oldani, Stéphanie Lacotte, Christian Toso

**Affiliations:** a Department of Visceral Surgery, Geneva University Hospital, Geneva, Switzerland; b Laboratory of Transplantation and Hepatology, University of Geneva, Geneva, Switzerland

**Keywords:** FGF21, Fertility, Non-alcoholic fatty liver disease, High-fat diet

## Abstract

**Objective:**

Obesity and associated liver disease are a growing public health concern. Pharmacological agents to treat non-alcoholic fatty liver disease are limited. FGF21, a hormone secreted by the liver and potent metabolic modulator, is a promising therapeutic target for this indication with several analogs currently in clinical development. However, concerns about a negative effect of FGF21 on female fertility have not been fully addressed.

**Methods:**

After induction of obesity, female C57BL/6N mice received a 7-day course of subcutaneously administered FGF21. Control groups received either high-fat diet (HFD) or a normal diet (ND). The mothers were then mated with lean males for 12 weeks. The estrous cycle was recorded for two weeks after breeding. The metabolic phenotype, liver steatosis and reproductive organs were assessed at sacrifice 14 weeks after treatment.

**Results:**

A short-course treatment of FGF21 leads to weight reduction during treatment but has no long-term impact on liver steatosis. A treatment with FGF21 leads to a reduction in the number of pregnancies (0 vs 1, *p* = 0.019) and no viable pup was born to a mother previously treated with FGF21. The FGF21 treatment affected the number of cycles (1 vs 3, *p* = 0.048) and amount in diestrus (54 vs 75%, *p* = 0.008) 12 weeks after the treatment. Additionally, the number of corpora lutea (0.8 vs 3.0, *p* = 0.016), and mature follicles (0 vs 1, *p* = 0.037) was reduced compared to the ND group while uterine histology remained unaffected.

**Conclusion:**

A short-term treatment with FGF21 has a long-term effect on female fertility in mice. This represents a potential safety concern for FGF21 analogs currently in clinical development. Reproductive health outcomes should be included in upcoming clinical trials.

## 1 Introduction

The global obesity epidemic fuels metabolic disorders and leads to a constantly increasing rate in non-alcoholic fatty liver disease (NAFLD), a disease that affects a quarter of the global population [1]. While some patients with NAFLD are completely asymptomatic, up to 40% eventually experience disease progression with the development of non-alcoholic steatohepatitis [2] and another 20% of these patients develop end stage liver disease and cirrhosis [3]. NAFLD also increases the risk of developing hepatocellular carcinoma ten-fold [4], one out of two patients with NAFLD cirrhosis develops hepatocellular cancer over a ten year period [5]. Surprisingly, NAFLD is also one of the most prevalent chronic disease with limited pharmacological therapies.

A multitude of ongoing clinical trials evaluate drug candidates for NAFLD [6]. One promising approach, with several molecules in late stage clinical development, is the treatment with endocrine fibroblast growth factor (FGF) analogs [7, 8]. One member of this family, FGF21, seems particularly interesting. This endogenous hormone exerts several beneficial metabolic effects; it increases β-oxidation of fatty acids, improves insulin resistance, stimulates the production of adiponectin and curbs energy expenditure. Efruxifermin, a fusion protein between a human IgG heavy chain linked to a modified human FGF21, decreased the relative hepatic fat fraction ≥50% in 88% of the treated patients vs no change in the placebo group [9]. Another FGF21 analog, BIO89-100, achieved a significant reduction in hepatic fat mass and markers of liver injury in a phase 1b/2a randomized clinical trial [10]. FGF21 analogs seem generally well tolerated with few short-term side effects. Diarrhea and increased appetite were the most common side effects but occurred in less than 20% of patients [9, 10, 11].

Nevertheless, concerns have been raised about the long-term safety of FGF21 analogs, especially with regards to its effect on bone mass and fertility [12, 13]. The published data on the effect of FGF21 on bone mass is conflicting. Several studies have shown that FGF21 transgenic mice and mice treated with recombinant FGF21 have reduced bone mass [14, 15, 16], while other authors were unable to find an effect of FGF21 on the bone [17]. The same inconsistencies are found in human studies [18, 19, 20]. The same controversies exist regarding the effect of FGF21 on female fertility. Early reports found that FGF21 induces infertility by inducing a starvation-like state and acting on the neuroendocrine axis [21, 22]. A later report suggested that this effect was mainly due to a hyper metabolism induced by FGF21 and that fertility could be restored by a high-fat diet (HFD) [23]. Overall, the effect of FGF21 on fertility remains unclear with sparse preclinical evidence. Additionally, no human data exists on this topic despite the multitude of clinical studies with FGF21 agonists and analogues [24].

The purpose of our study is to evaluate the long-term effect of FGF21 on female fertility in a murine model. This reproduces a clinical scenario where patients suffering from obesity induced liver disease receive a treatment with a FGF21 analogue and might wish to conceive later. Our study sheds light on this important, albeit understudied side effect of FGF21 analogues in a patient population that is already affected by decreased fertility [25]. In our study, we used a model of diet-induced obesity and non-alcoholic fatty liver disease to study the effect of subcutaneously administered FGF21 on long-term fertility.

## 2 Materials and methods

### 2.1 Animal experimentation

Animal experimentation was carried out under the terms of an experimental protocol approved by the ethical committee of the University of Geneva and the veterinary authorities of the canton Geneva (GE85-21). All mice were housed in the animal facility of the University of Geneva under 12/12-hour light/dark cycles with free access to food and water. Five-week-old dams (C57BL/6N) were purchased from Charles River Laboratories (Ecully, France). They were then randomly assigned to a control diet (ND: 17% kcal fat, 61% kcal carbohydrate, 22% kcal protein; Envigo TD.120455) or an energy-rich Western style high fat diet (HFD: 45% kcal fat, 41% kcal carbohydrate, 15% kcal protein; Envigo TD.08811). After 12 weeks on their respective diets, the female mice were mated with C57BL/6N males on control diet. Throughout mating, pregnancy and lactation, dams received the same diet as before mating (ND or HFD).

### 2.2 Subcutaneous administration of FGF21

Recombinant human FGF21 (Cat No. CYT474) was purchased from Prospec Protein Specialist (Ness Ziona, Israel). The FGF21 was dissolved in isotonic saline solution NaCl 0.9% and administered subcutaneously through an osmotic pump in a continuous fashion in a dose of 1 mg/kg/d. For this, we implanted a 7-day osmotic pump (Alzet osmotic pump, Durtec Corp., Cupertino, USA). The pumps were inserted subcutaneously through a small incision in the loose skin over the neck (closed with separated Prolene 6-0 sutures). After seven days, we removed the osmotic pump and checked that the whole volume had been administered.

### 2.3 Experimental design

**Figure 1:**
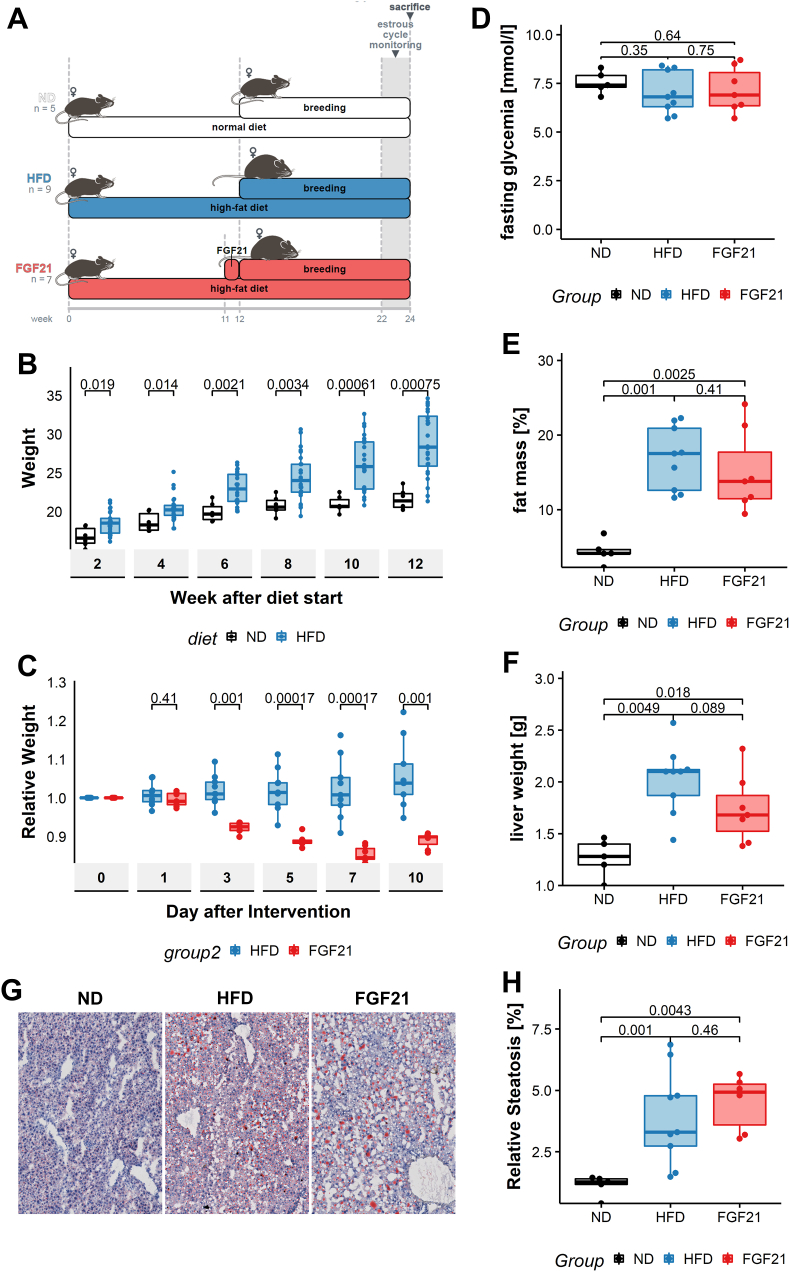
**FGF21 has no effect on the metabolic phenotype in the long term**. Female C57BL6 mice were fed ad libitum with either high fat (HFD) diet or normal diet (ND) during 12 weeks. Animals in the FGF21 group received a 1-week course of subcutaneous FGF21 before the start of breeding (A) Experimental setup. (B) Weight was recorded weekly before breeding. (C) Weight was recorded daily during the treatment with FGF21 (D) Before sacrifice mice were fasted for 6h and blood glucose levels were measured. (E) Fat-mass was measured by echo-MRI before sacrifice (F) Liver weight of the dissected liver was determined after sacrifice. (G) Snap frozen liver samples from all groups were stained with Oil-Red-O and counterstained with hematoxylin, representative images are shown. (H) The total surface of the liver occupied by steatosis was quantified with bioimaging analysis tools. Statistical analysis: Wilcoxon rank-sum test (B, C, D, E, F, H). Median with interquartile ranges are displayed. ND n = 5, HFD n = 9, FGF21 n = 7.

To assess the influence of subcutaneously administered FGF21 on female fertility we included three different experimental groups. One control group that received normal diet before and during mating (named ND). Another control group that received Western style diet twelve weeks before and throughout mating (named HFD). Finally, an interventional group received twelve weeks of Western style diet before and throughout mating. Additionally, these mothers received recombinant human FGF21 for seven days immediately before mating (named FGF21) (Figure 1A).

### 2.4 Measures of fertility

The pregnancy status was assessed multiple times per week through visual inspection of the dams, weight recording [26] and the examination of the vaginal opening [27]. The number of pregnancies was defined as the total number of pregnancies per dam during the entire mating period. We defined the total number of pups per dam as the number of pups counted by the animal caretaker in the cage immediately after birth. We defined the number of pups alive as the number of pups per dam that survived beyond day three of life.

### 2.5 Assessment of reproductive cycle

At the end of the mating period the estrous cycle, which refers to the reproductive cycle in rodents, was recorded continuously over two weeks. The normal estrous cycle lasts between four and five days and is divided in four phases, namely proestrus, estrus, metestrus and diestrus. Each phase is characterized by a distinctive vaginal smear. We performed a vaginal smear daily to determine the estrous phase of each dam in the following manner [27].

The animal was manually restrained, and the tail elevated to visualize and assess the vagina. The vaginal cells were flushed by gently introducing approximately 80 μl of normal saline 0.9% using a pipette. We slowly released the liquid into the vaginal opening without penetrating and drew it back into the tip; this is repeated about 4–5 times with the same tip. The fluid containing few drops of cell suspension was introduced on a glass slide, and immediately examined under a light microscope.

We determined the phase of the cycle according to the proportion of nucleated epithelial cells, cornified epithelial cells and leukocytes in the vaginal smear [28]. In the proestrus phase mostly nucleated and some cornified epithelial cells are present in the smear, some leukocytes might still be visible in the early proestrus phase. During the estrus phase the smear is dominated by cornified epithelial cells. If the cycle is not interrupted by a pregnancy the next phase is metestrus which is defined by a presence of cornified epithelial cells, nucleated epithelial cells and some leukocytes. The cycle then progresses to the diestrus phase, the longest phase of the cycle, where the dominant cell type in the smear are leukocytes.

### 2.6 Sacrifice

All dams were sacrificed at the end of the mating period and after having monitored the estrous cycle for two weeks. Dams were sacrificed in the diestrus phase of the estrous cycle by exsanguination under inhaled isoflurane anesthesia. Blood was collected by puncture of the inferior vena cava, liver, uterus and ovaries were dissected and weighed. Liver samples were snap frozen and stored at -80 °C until further use. The rest of the liver was placed in buffered 4% formaldehyde solution for later histological analysis.

### 2.7 Histology

Snap frozen liver specimens were sectioned at 6 μm and stained with Oil Red O and counterstained with hematoxylin to visualize hepatic lipid accumulation. Additionally, uterus and ovaries were fixed in buffered 4% formaldehyde solution and paraffin embedded before sectioning. All sections were stained with hematoxylin and eosin. The number of corpora lutea was manually and blindly counted for both ovaries of each animal and normalized per ovary. The total surface of the corpora lutea was quantified with the bioimage analysis software QuPath [29]. The endometrial thickness was measured at three different points and the mean was chosen for reporting.

### 2.8 Statistical analysis

Statistical analyses were performed using R and R Studio [30, 31]. Continuous outcomes were compared using the Wilcoxon rank-sum test. Proportions between more than two groups were compared with the Pearson's chi-squared test. The limit for statistical significance was set at *p* = 0.05. All measures were expressed as median.

## 3 Results

### 3.1 Short course treatment by FGF21 does not have a long-term effect on metabolism or steatosis

The mothers fed a HFD gained more weight than mothers fed a ND and were significantly heavier from week 2 after the start of the diet (18.3 vs. 16.5 g, *p* = 0.019) to week 12 before the intervention (28.3 vs. 21.4, *p* = <0.001) (Figure 1B).. Continuous subcutaneous administration of FGF21 induced weight loss without reducing food intake [32, 33]. We observed a weight loss of 15.5% in animals treated with FGF21 at the end of the treatment period at day 7, compared to a weight gain of 1% in those without treatment (*p* = <0.001). This weight loss is in line with previous findings and demonstrates the potent short-term metabolic impact of FGF21 [32] (Figure 1C). In the long-term, 14 weeks after treatment, we were unable to detect metabolic differences between animals that received FGF21 and the HFD control group concerning fasting glycaemia or fat mass measured by Echo MRI (Figure 1D, E). We observed a trend towards decreased liver weight at sacrifice (1.68 vs 2.08 g, *p =* 0.089) (Figure 1F). However, this did not translate in a lower rate of steatosis for animals that received FGF21. Animals on HFD had an increased relative tissue surface of steatosis compared to animals on ND (3.29 vs 1.27%, *p*=<0.001), while the treatment with FGF21 had no effect on steatosis in the long-term (4.93 vs 3.29%, *p* = 0.46) (Figure 1G, H).

### 3.2 FGF21 reduces long-term fertility

**Figure 2:**
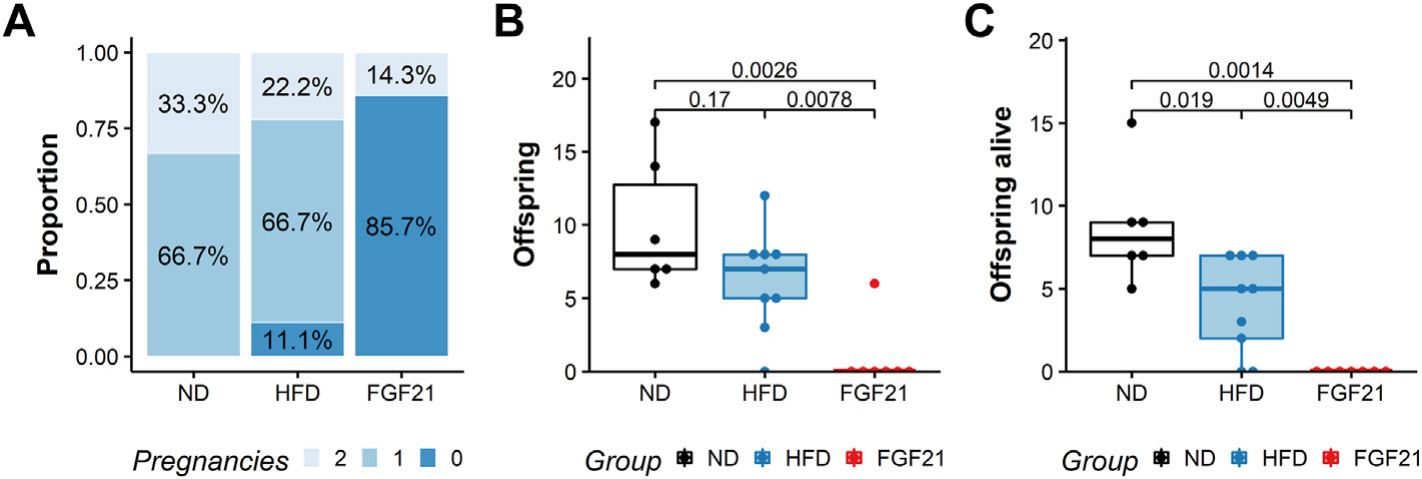
**A short-course FGF21 treatment has a lasting impact on female fertility**. 17-week old female C57BL/6N having previously received 12 weeks of normal diet (ND), high-fat diet (HFD) or HFD and a 1-week course of subcutaneous FGF21 (FGF21) were mated with lean males for a twelve-week period. (A) The pregnancy status was assessed several times weekly by visual inspection, weight change and assessment of vaginal opening. This panel represents the proportion of females that were pregnant, 0, 1 or 2 times during the mating period. (B) The number of offspring born per mother over the whole twelve-week period was recorded immediately after birth. (C) The number of offspring alive at day three after birth was recorded over the whole breeding period. Statistical analysis: Wilcoxon rank-sum test (A, B, C). Median with interquartile ranges are displayed. ND n = 5, HFD n = 9, FGF21 n = 7.

Overweight mothers who received a subcutaneous administration of recombinant human FGF21 before mating showed decreased fertility. We observed that mothers fed a HFD and treated with FGF21 presented a significantly lower number of pregnancies compared to mothers only receiving a HFD (0 vs 1, *p* = 0.019) (Figure 2A). Similarly treatment with FGF21 significantly decreased the number of pups born compared to mothers who received HFD without FGF21 (0 vs 6, *p* = 0.012) (Figure 2B). Pups born to HFD mothers suffered from a higher perinatal mortality compared to ND controls, illustrated by a significantly lower number of pups alive at day three of life (4.5 vs 7, *p* = 0.0011). This effect was more pronounced in mothers who received an FGF21 treatment than in mothers with only a HFD (0 vs 4.5, *p* = 0.0082). No pup born to any of the eight mothers in the FGF21 group survived until day three.

### 3.3 FGF21 induces alterations in the estrous cycle in the long term

**Figure 3:**
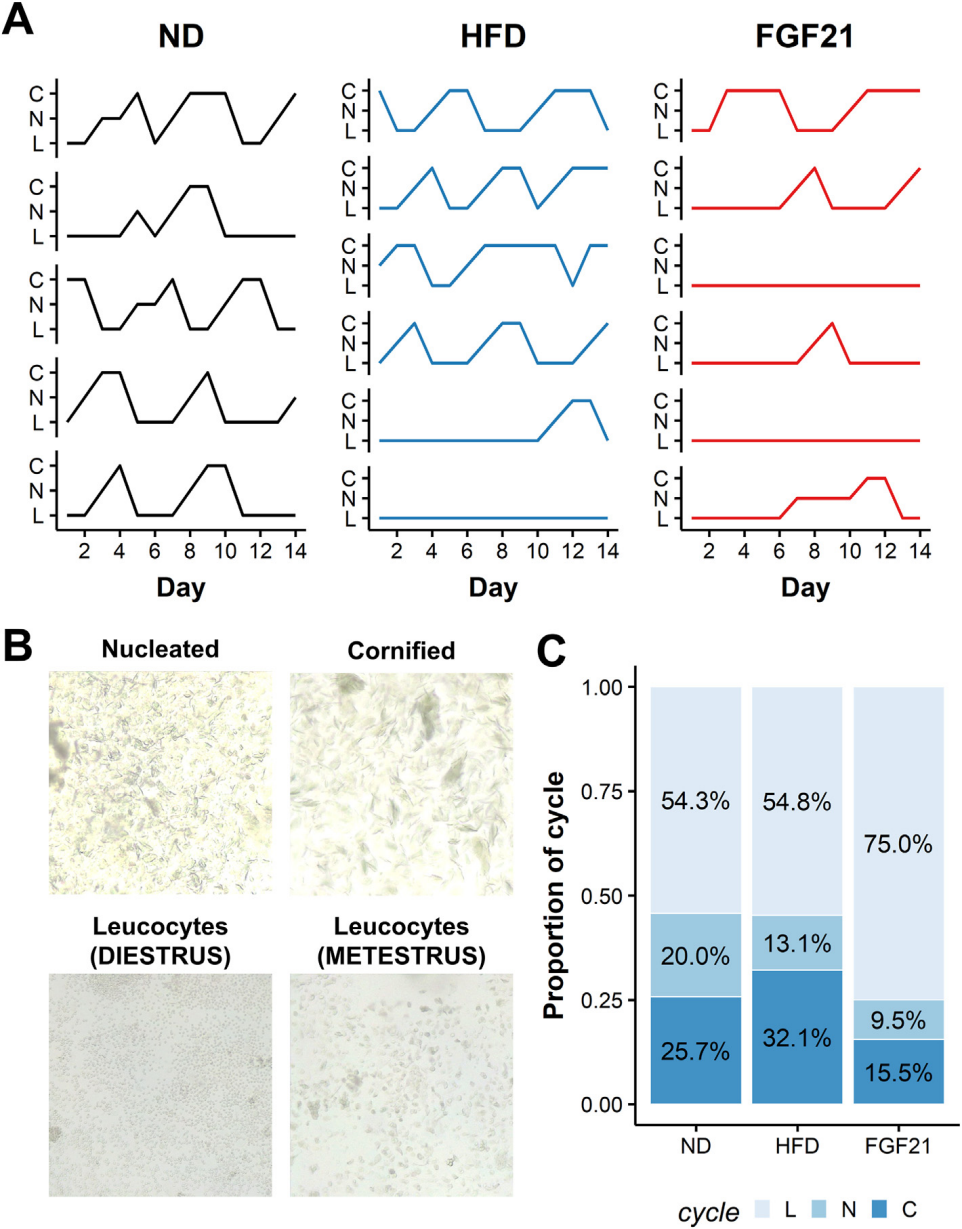
**FGF21 disrupts the estrous cycle in mice**. At the end of the breeding 12-week period the estrous cycle was recorded each day by vaginal cytology. Animals having previously received a normal diet (ND), high-fat diet (HFD) or HFD and a 1-week course of subcutaneous FGF21 (FGF21) were included. (A) The estrus stage was recorded for each animal and day according to the vaginal cytology. (B) Representative images for each stage of the cycle are included, a majority of nucleated epithelial cells are present in proestrus, cornified epithelial cells dominate in estrus, leucocytes are predominant in diestrus and leucocytes with the other two cell types in metestrus. (C) The proportion of time spent in each of the stage is represented in percentage. Abbreviations: L = leucocytes predominant (diestrus or metestrus), N = nucleated epithelial cells are predominant (proestrus), C = cornified epithelial cells are predominant (estrous).

To assess if the decreased fertility was associated with alterations of the estrous cycle, we performed daily vaginal cytology for two weeks after the breeding period and before the sacrifice (Figure 1A). The mouse estrous cycle, the rodent equivalent to the human menstrual cycle, typically lasts between four and five days. Each stage of the cycle can be determined by the proportion of nucleated epithelial cells, cornified epithelial cells and leucocytes (Figure 3B) [34]. Each mother in the ND group exhibited a normal cycle with a duration between 4-5 days and an average of 2.4 cycles over the 14-day assessment period. In the HFD group, all animals exhibited an estrous cycle, except for one mother. Each mother had on average 2 cycles, with a trend towards a longer estrus phase as described recently by a different group (2 vs 1.67 days, *p* = 0.14) [35]. In the FGF21 group, not only did we observe a decreased number of cycles compared to the ND group (1 vs 3, *p* = 0.048) but also most observed cycles deviated from the typical 4–5 days per cycle (Figure 3A). The proportion of days spent in either diestrus, metestrus (54 vs 54 vs 75%, *p* = 0.008) or estrus (25 vs 32 vs 15%, *p* = 0.040) was significantly different between the three groups (Figure 3C).

### 3.4 FGF21 reduces the number of ovulations but induces no structural changes to the reproductive organs

**Figure 4:**
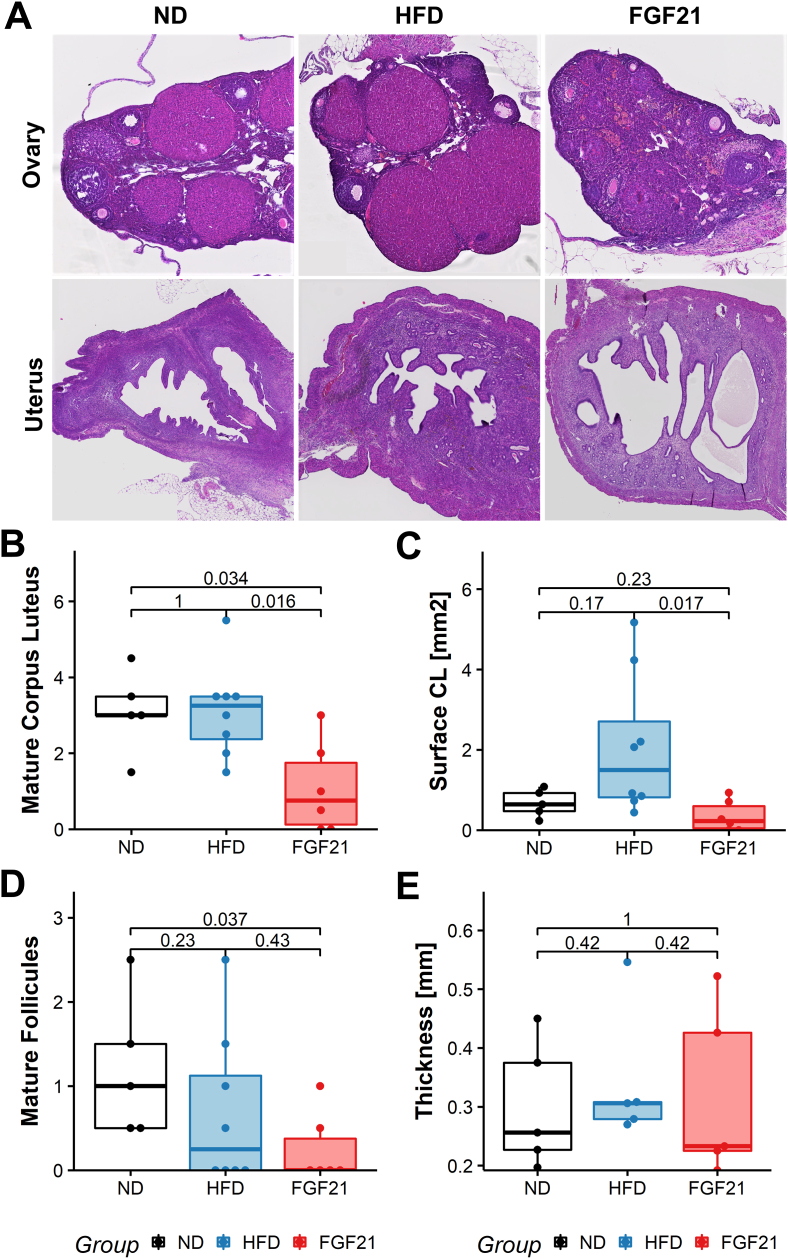
**A short-course of FGF21 treatment reduces the number of corpora lutea and mature follicles**. Animals were sacrificed in diestrus and reproductive histology was assessed. Animals having previously received a normal diet (ND), high-fat diet (HFD) or HFD and a 1 week course of subcutaneous FGF21 14 weeks prior (FGF21) are included. (A) Representative histology of uterus and ovary of one animal per group is shown. (B) The mean number of corpora lutea per animal is displayed. (C) The mean total surface occupied by corpora lutea by animal in mm^2^ is shown (D) The mean number of mature Graafian follicles per animal is displayed. (E) The mean endometrial thickness per animal measured perpendicularly to the serous surface at three different points is displayed. Statistical analysis: Wilcoxon rank-sum test (B, C, D, E). Median with interquartile ranges are displayed. ND n = 5, HFD n = 8, FGF21 n = 6.

Next, we wanted to assess if treatment with FGF21 induced structural changes in the reproductive organs that could explain the decreased fertility. We found that a subcutaneous treatment with FGF21 decreased the number (0.75 vs 3.25, *p* = 0.016) and the total surface of corpora lutea (CL) (0.225 vs 1.49 mm^2^, *p* = 0.017) 14 weeks after the treatment compared to the HFD control group (Figures 4A, 4B, 4C). The CL persist after ovulation and the number of CL per ovary is therefore representative for the number of ova shed [36]. Together with the decrease in the number of mature Graafian follicles in the FGF21 group compared to the ND group (0 vs 1, *p* = 0.037) (Figure 4D) this is further confirmation for the decreased ovulation rate and the long-term negative impact of FGF21 on fertility. We did not detect any abnormalities of uterine morphology (Figure 4E).

## 4 Discussion

FGF21 analogs and agonists are a major target for the treatment of NAFLD with several molecules in late stage clinical development and promising results on the reduction of steatosis and inflammation [8, 9, 10]. Concerns about the safety profile with a negative impact on bone density and female fertility have previously been raised but not consistently confirmed [14, 16, 21]. In contrast to previous studies, we found that a short course of subcutaneously administered FGF21 negatively affected female fertility in the long-term and was not rescued by HFD. This to a point where eight mothers treated with FGF21 did not give birth to any viable offspring over a 12-week breeding period.

An initial study by Owen et al. found that transgenic mice continuously overexpressing FGF21 are infertile [21]. In contrast to their study, we only treated the mothers in the FGF21 group for one week with a subcutaneously administered exogenous FGF21 but found very similar effects 14 weeks after the treatment was stopped. As described by Owen et al. we found decreased fertility, perturbed ovulatory cycles, the presence of mature Graafian follicles but a decreased number of corpora lutea. Still in transgenic mice overexpressing FGF21, Singhal et al. reported that HFD partially restores fertility in FGF21 transgenic mice, 75% of FGF21 transgenic mothers on HFD became pregnant compared to none in the FGF21 transgenic control group on ND [23]. However, the authors did not include a comparison of fertility outcomes between FGF21 transgenic and WT mice. In our study, only 14% of females that received exogenous FGF21 prior to breeding became pregnant, significantly less than in both control groups. The main strengths of our study compared to the two previous reports is the use of a treatment modality close to the clinical reality with the subcutaneous administration of FGF21 and that we studied the long-term effect on fertility of the drug.

Previous studies have shown that some of the metabolic effects of FGF21 such as increased energy expenditure or glucose homeostasis are mediated through the central nervous system [37, 38]. More specifically FGF21 receptors in the hypothalamus are implicated in the regulation of taste preference, systemic glucocorticoid levels and suppress physical activity [39, 40]. Similarly Owen et al have shown that hypothalamic neurons in mice overexpressing FGF21 are unable to induce a gonadotropin-releasing hormone release in response to a surge of estradiol and therefore fail to produce the luteinizing hormone surge required for ovulation. This mechanistic explanation is compatible with the hypogonadic phenotype we observed with a decreased number and size of corpora lutea and disturbed ovulatory cycles [21]. Nevertheless, it is surprising that FGF21 with a half-life of only 0.5–2 h induced persistent reproductive changes up to 14 weeks after the last administration of the drug, while we were unable to detect any metabolic effect nor impact on liver steatosis at that time point (Figure 1) [41].

FGF21 also acts centrally in the suprachiasmatic nucleus (SCN) of the hypothalamus to modulate circadian behavior. Bookout et al. showed that transgenic overexpression of FGF21 reduced overall activity but increased activity during rest phase through a decreased output of the inhibitory neuropeptide vasopressin from the SCN [40]. At the same time, vasopressin plays an important role in maintaining regular ovulation through the hypothalamic estrogen kisspeptin signaling cascade [21, 42, 43]. The long-term disruption of the circadian rhythm by a short term administration of exogenous FGF21 might have contributed to the decreased fertility we observed in our study.

We also observed a negative effect of HFD alone on fertility, although somewhat milder than previously reported [44, 45, 46]. While the median number of pregnancies was unaffected by the diet, obesity and HFD significantly decreased the number of pups alive at day three of life. In contrast to previous studies, HFD did not affect the number of CL.

To allow for a well-developed obesity phenotype we kept the mice for twelve weeks on ND or HFD before starting the mating. For this reason, we started mating not until week 17 of life and sacrificed the animals at week 31. The relatively advanced age might have contributed to the decrease in fertility observed. Nevertheless, all groups were of equal age and large scale studies have shown that the pregnancy success rate remains unaffected in C57BL6 mice up to 12 months of age [47].

Our study highlights the negative impact of FGF21 on long-term female fertility in mice. This represents a reproductive health safety concern for the FGF21 analogs that are currently in clinical development. The inclusion of reproductive health outcomes in the clinical trial design for upcoming clinical trials could provide valuable human data and should be considered.

## 5 Conclusion

Our findings mandate further research on the effect of FGF21 on female fertility. Several FGF21 analogs are in clinical development for the treatment of obesity associated NAFLD. Obesity by itself already reduces female fertility. If the mechanisms of long-term fertility impairment are shown to be translatable to humans, this may have implications for the clinical use of FGF21 therapy. Our data suggest that assessing female fertility should be priority in the safety evaluation of FGF21 analogs and agonists already in clinical development.

## Declarations

### Author contribution statement

Beat Moeckli and Stephanie Lacotte: Conceived and designed the experiments; Performed the experiments; Analyzed and interpreted the data; Wrote the paper.

Thuy-Vi Pham: Performed the experiments; Analyzed and interpreted the data; Wrote the paper.

Christian Toso: Conceived and designed the experiments; Wrote the paper.

Florence Slits: Performed the experiments.

Samuel Latrille: Performed the experiments; Wrote the paper.

Andrea Peloso and Vaihere Delaune: Analyzed and interpreted the data; Wrote the paper.

Graziano Oldani: Analyzed and interpreted the data.

### Funding statement

Christian Toso was supported by Schweizerischer Nationalfonds zur Förderung der Wissenschaftlichen Forschung [182471], Fondation Leenaards [5489], Fondation Marie et Francis Minkoff.

### Data availability statement

Data associated with this study has been deposited at https://github.com/moecklib.

### Declaration of interest's statement

The authors declare no conflict of interest.

### Additional information

No additional information is available for this paper.
